# Innovations in Corneal Transplantation: The Role of Eye Banks, Donor Corneas, and Artificial Alternatives in Combating Worldwide Corneal Blindness

**DOI:** 10.22336/rjo.2026.03

**Published:** 2026

**Authors:** Mahesh Chandra, Jitendra Singh, Gaurav Dubey, Govind Gaurav Pandey, Ayush Jha

**Affiliations:** 1 Department of Optometry, Dr. Sushila Tewari Hospital & Government Medical College, Haldwani, Uttarakhand, India; 2 Clear Vision Eye Care Centre, Faridabad, Haryana, India; 3 Department of Optometry, Uttar Pradesh University of Medical Sciences, Saifai, Etawah, Uttar Pradesh, India; 4 Arjun Eye Bank, Dr. Sushila Tewari Hospital & Government Medical College, Haldwani, Uttarakhand, India; 5 Jeewan Dan Medical Centre, Moti Nagar, Haldwani, Uttarakhand, India

**Keywords:** National Programme for Control of Blindness (NPCB), Eye Bank Training Centre (EBTC), Eye Donation Centres (EDCs), Eye Bank Association of India (EBAI), National Accreditation Board of Hospitals (NABH), Hospital Cornea Retrieval Program (HCRP), Penetrating Keratoplasty (PK), Deep Anterior Lamellar Keratoplasty (DALK), Endothelial Keratoplasty (EK), CORE = Corneal Opacity Rural Epidemiological Study, DALK = Deep Anterior Lamellar Keratoplasty, DMEK = Descemet’s Membrane Endothelial Keratoplasty, DSAEK = Descemet’s Stripping Automated Endothelial Keratoplasty, DSEK = Descemet’s Stripping Endothelial Keratoplasty, EB = Eye Bank, EBTC = Eye Bank Training Centre, EBAI = Eye Bank Association of India, EDCs = Eye Donation Centres, EK = Endothelial Keratoplasty, HCRP = Hospital Cornea Retrieval Program, MK Medium = McCarey–Kaufman Medium, NABH = National Accreditation Board for Hospitals, NPCB = National Programme for Control of Blindness, OOKP = Osteo-Odonto-Keratoprosthesis, PK = Penetrating Keratoplasty

## Abstract

**Aim:**

Corneal disorders—including degeneration, dystrophy, infection, and inflammation—rank as the fifth leading cause of global visual impairment. In developing regions, conditions such as trachoma and xerophthalmia further increase this burden. This review explores the current landscape, challenges, and advancements in corneal blindness management, with a focus on the role of eye banks and keratoplasty techniques.

**Objectives:**

(i) Assess global burden and causes of corneal blindness; (ii) Highlight eye banks’ roles in donor tissue management; (iii) Identify challenges in eye banking and transplantation; (iv) Evaluate keratoplasty procedures; (v) Examine surgical innovations and therapies such as corneal collagen cross-linking.

**Methodology:**

A comprehensive literature review was conducted, analyzing peer-reviewed publications, health reports, and clinical guidelines. Data from both developed and developing countries were considered to provide a holistic perspective.

**Discussion:**

Corneal transplantation remains the most effective treatment for advanced corneal blindness; however, a significant gap persists between the demand for donor corneas and their availability. Advances in preservation techniques and the shift toward lamellar keratoplasty have improved graft survival and visual outcomes. Nevertheless, limited donor awareness and infrastructural constraints continue to affect successful implementation.

**Conclusion:**

Advanced keratoplasty methods—Penetrating Keratoplasty, Deep Anterior Lamellar Keratoplasty, and Endothelial Keratoplasty—have improved outcomes by reducing rejection and accelerating recovery. The adoption of femtosecond laser technology has enhanced surgical precision. Corneal collagen cross-linking now offers a minimally invasive option to delay or prevent transplant in ectatic conditions. Despite these advancements, global eye banks face persistent challenges in donor recruitment, tissue preservation, and ethical management. Continued innovation, improved infrastructure, and strengthened public health efforts are crucial to overcoming these barriers and reducing the global burden of corneal blindness.

## Introduction

The concept of eye donation is deeply rooted in India’s cultural heritage, exemplified by the legend of Kannapa Nayanar, a Shaivite saint who, in pure devotion, offered both his eyes to Lord Shiva. This story symbolizes the selflessness and compassion central to eye donation, highlighting its enduring spiritual and societal significance in transforming lives through corneal transplants [1].

Vladimir Filatov (1930) is viewed as the father of eye banking, as he conceptualized saving the whole eye globe for later use in keratoplasty. Before his work, Guillaume Pellier de Quengsy introduced the idea of corneal transplantation in the 18th century, and Eduard Zirm successfully performed the first corneal transplantation in 1905. The first reported eye bank was established by Dr. R. Townley Paton and Dr. John MacLean (1940), and the first reported sight-rebuilding in New York was in 1944 [2,3].

In 1905, Eduard Zirm established the first eye bank (EB) and performed the first successful corneal transplant. As mentioned by Dr. R.E.S. Muthiah, the Regional Institute of Ophthalmology, Chennai, was the first Indian EB in 1945, and the first corneal transplant was performed in 1948. Prof. R.P. Dhanda (1960-Indore) performed his first corneal transplant, alongside Dr. Kalevar, before 1970, and later performed various corneal transplants at the Civil Hospital, Ahmedabad (Gujarat) [4].

Corneal blindness is the third leading cause of blindness globally, affecting around 10 million people with bilateral corneal blindness. Data from India’s Rapid Assessment of Avoidable Blindness survey reveals that 37.5% of corneal blindness cases occur in individuals aged up to 49, while 7.4% affect those over 50 [5,6].

Gain et al. (2012) estimated that 284,000 donated corneas were globally available across 82 countries, with approximately 185,000 transplanted [5]. India has approximately 1.2 million people affected by corneal blindness, with an additional 25,000 to 30,000 new cases emerging annually. According to the Corneal Opacity Rural Epidemiological study (CORE), the leading causes of corneal visual impairment are bullous keratopathy (46.2%), corneal dystrophy and degeneration (23.1%), and trachoma (15.4%). Another study highlighted keratitis as a major contributor, with 36.7% of cases occurring in childhood, 28% due to trauma, and 17.7% in adulthood [7-9].

An eye bank is a nonprofit organization that recovers, screens, and preserves corneas for transplantation, ensuring safety and quality while adhering to strict ethical standards. It supplies tissue to transplant centers as part of an organ network [10,11]. Indian eye banks operate a three-tier, integrated system to ensure that safe, high-quality corneal tissue reaches every recipient efficiently and cost-effectively. They receive support from prominent global health nonprofits such as Orbis, SightLife, and Eyesight International. Recently, the Indian Eye Bank was also accredited by the National Accreditation Board for Hospitals (NABH) [12,13].

In 1976, the National Programme for Control of Blindness (NPCB) against blindness was introduced by the Ministry of Health and Family Welfare, led by Dr. Govindappa Venkataswamy, Dr. Lalit Prakash Agarwal, and Sir John Wilson. In 1985, the national fortnight eye donation awareness programme (August 26–September 8) was initiated. The Eye Bank Association of India (EBAI), established in 1989, supports this mission by enhancing awareness and standardizing eye banking practices nationwide [4,14-16].

The Human Organs Act of 1994 replaced the 1983 Corneal Transplant Act, enabling eye harvesting by registered practitioners. In 2004, the National Programme for Control of Blindness (NPCB) partnered with Non-Governmental organizations (NGOs) to launch the National Plan for Corneal Blindness, which supported 82 eye banks and 177 Eye Donation Centers (EDCs) with Rs 2000 per pair of eyes collected. Government eye banks also received Rs 25 lakh each to improve quality, recruit staff, and train grief counsellors and doctors [10,13,14,16-19].

In 2012, the Eye Bank Association of India (EBAI) partnered with SightLife to enhance tissue availability. This collaboration linked eye banks with corneal surgeons in 50 cities, increasing tissue collection by 30% annually, around 50,000 eyes. Eye Bank Association of India (EBAI) also introduced guidelines for safety standards, training, infrastructure, and other requirements to ensure uniform quality across the country [13,20,21].

### Research gap

Although keratoplasty has achieved substantial success in restoring vision, there remains a critical gap in consolidated data on its real-world implementation, operational challenges, and accessibility in low- to middle-income countries. While technological advancements such as femtosecond laser-assisted keratoplasty and endothelial procedures have improved outcomes, their availability in resource-constrained regions remains limited and underreported. Moreover, the socio-cultural, ethical, and legislative factors affecting corneal donation rates remain inadequately addressed in current literature. This review aims to bridge these information gaps and provide evidence-based insights to guide clinical practice and policy-making.

### The infrastructure of the eye bank

India’s eye banking system involves three key entities: Eye Bank Training Centre (EBTC - responsible for scheduling functions and training), Eye Bank (often at medical colleges or tertiary centers), and the Eye Retrieval Centers (ERC), focused on eye donation awareness, donor coordination, tissue harvesting, and serological testing. Eye banks handle public awareness, tissue processing, preservation, evaluation, and distribution to keratoplasty centers [22].

According to the National Programme for Control of Blindness (NPCB), India has 435 operational eye banks and Eye Donation Centers (EDCs), with 1 eye bank or EDC per 3 million people. The corneal collection rate in India was 63,256 in 2016-17, a significant increase from 38,746 in 2007-08. Tamil Nadu led in corneal collection among Indian states. However, low literacy rates and rural residence contribute to lower corneal donation rates [23-25].

The 12th Five-Year Plan (2012–17) allocated a significant budget for corneal donation, preservation, and distribution. The Hospital Cornea Retrieval Program (HCRP), launched in 1990, promotes corneal donation by targeting hospital-based deaths and providing grief counselling to families. The Hospital Cornea Retrieval Program (HCRP) benefits young donors by providing detailed medical histories, faster tissue retrieval, and better access to medical information [16,26,27].

The National Eye Bank at AIIMS, New Delhi, has also run nonprofit eye banking services for the last five decades, leading the development of the eye banking improvement policy in India [28].

Eye banks need essential facilities for 24/7 operations, including landlines, mobile phones, and internet access; reliable transport; refrigeration for tissue and blood samples; sterilization equipment; a serological lab; and preservation facilities. Required ophthalmic instruments for corneal retrieval include a laminar flow hood, a specular microscope, and a slit lamp. Requirements for the surgical procedure include a torch, loupe, drape, syringes, needles, blood-collection vials, specula, surgical blades, corneal scissors, iris forceps, artery forceps, muscle hooks, enucleating spoons, and strabismus scissors [22].

An efficient eye bank requires an experienced corneal surgeon as its director, responsible for overseeing all operations; a skilled medical team to handle tissue collection, evaluation, preservation, and distribution; public awareness programs; and collaboration with government and health agencies [10,29].

India’s future in eye banking holds great potential. To bridge the gap between supply and demand for donor corneas, the focus must be on improving infrastructure, training, human resources, and resource management. In November 2017, the National Expert Group at AIIMS, New Delhi, recommended steps to enhance eye banking and address corneal blindness, which is crucial for strengthening the system and meeting the increasing need for corneal transplants [30].

India has the potential to create a world-class Eye Bank and corneal transplantation network supported by top medical institutions, skilled surgeons, and advanced healthcare technologies. Achieving this requires a national movement focused on raising awareness, improving eye bank infrastructure, streamlining corneal collection and distribution, and ensuring equitable access to transplants [30].

A robust public-private partnership could advance this initiative by leveraging government healthcare programs alongside the private sector’s efficiency and innovation. Comprehensive national policies on training, quality standards, and funding are essential to meet the increasing demand for corneal transplants and reduce corneal blindness. By engaging communities, enhancing public participation, and fostering collaboration between medical professionals and government agencies, India’s eye banking system can transform into a global leader in vision restoration [30].

### Functions of the eye bank

- **Eye donation and appreciation** through the public education crusade come with the coordination of hospitals and community groups. The aim is to inform the public about the benefits and simplicity of eye donation by encouraging individuals to pledge to donate their eyes after death [22].
- **Eye Banks rigorously screen possible donors based on different factors like age, medical history, and risk of infectious diseases to ensure tissue quality. Upon confirming a donor’s eligibility, the eye bank retrieves the corneas shortly after death to maintain their viability** [30].
- **The received cornea is tested using advanced technology to determine the fairness of the cornea for transplantation. Tissues meeting stringent quality standards are used for transplantation; otherwise, they are used for research or education** [22].
- **For an efficient and fair distribution of the cornea, the eye bank coordinates with surgeons and hospitals to allocate corneas to patients according to different factors, such as medical urgency, compatibility, and other factors** [15].
- **Eye Banks contribute to the development of new treatments for eye diseases by providing tissues for research, medical education, and other eye care procedures** [22].

### Process of eye donation

Corneal donation does not depend on age, sex, blood group, refractive error, cataract surgery, or any other systemic disease; however, medically rejected corneas are used for medical research and studies. The cornea should be collected within 6 to 8 hours of the individual’s death; the procedure incurs no cost, yet there remains a global shortage of corneal donors. The recipient of the cornea is short-listed by the eye bank [31].

Voluntary eye donation is a social responsibility, and individuals should willingly come forward to pledge their eyes. Although pledge rates in India are rising, various factors can influence actual donations. Since donation decisions often need to be made immediately after a person’s death, the grieving process can make it challenging for family members to grant corneal donation. This underscores the importance of donation notices and coordination with eye banks. Volunteers and grief counsellors play a crucial role in bridging the gap between the community and eye banks, helping families make informed decisions during difficult times [32,33].

### Specimen sampling of the donor and infection control

Every donated tissue must be retained until thorough tests are performed to ensure the safe and optimal use of donated tissues in Eye Banks. The included screening tests are human immunodeficiency virus (HIV), hepatitis B surface antigen (HBsAg), hepatitis C virus (HCV), syphilis, and any other pertinent infectious diseases. Only after confirming non-reactive results should the ocular tissue be used [22].

Eye Banks operate under universal precautions to prevent health hazards. This includes mandatory hepatitis B and other suggested vaccinations for ground staff to minimize exposure risks. Detailed records are essential, documenting each piece of equipment’s maintenance, certification, sterilization, and cleaning protocols. All records are kept for at least three years, including measurement status for critical measuring instruments, to ensure all equipment is functioning correctly and safely [22].

### Preservation of the cornea

Early storage techniques for corneal tissue were basic, involving placing whole eyes in small glass containers under controlled humidity and temperature conditions. However, these methods provided a limited preservation window, necessitating transplantation within 48 hours of the donor’s death to ensure endothelial cell viability. Quality control relied primarily on corneal appearance and post-mortem time, without effective measures to screen for potential infectious diseases [26].

In the 1970s, McCarey and Kaufman introduced a significant improvement in preservation methods. They developed a technique using a low-level culture medium supplemented with dehydrating agents and antibiotics, allowing corneal tissue to remain viable for 7-10 days. This was a major advancement in extending storage times and preserving endothelial viability [34,35].

Today, advancements in storage techniques enable even longer preservation periods. For example, in organ culture conditions, corneal tissue can remain viable for up to 5 weeks. In India, the tropical climate poses unique challenges for endothelial viability, necessitating specialized solutions. Cornisol, developed by Aurolab in Madurai, is a notable innovation that enables two-week storage of corneas. Like Optisol GS, Cornisol offers an affordable option for intermediate storage. Previously, the McCarey-Kaufman (MK) medium was widely used as a cost-effective, short-term preservation solution, initially introduced by Ramayamma International Eye Bank (1994) and later adopted by other eye banks in India, such as Rotary Aravind International Eye Bank in Madurai [10,22,34].

Current trends also focus on minimizing the time from death to preservation to optimize endothelial cell viability. Techniques like corneoscleral rim excision (in situ) allow for efficient tissue preparation, further enhancing the quality and usability of the donated corneal tissue [4,12,17,36].

### Shipping of the tissue

The individually packed tissue is shipped in an aseptic container with a tamper-evident seal to maintain quality and prevent contamination. The tissue label on a container provides details until the tissue is used, and to maintain tissue quality during transit, the temperature is kept cool with wet ice or gel packs. Such a procedure accounts for different factors depending on transit time and temperature conditions [17,20,22].

### Recipient follow-up

The minimum required details for the recipient are the recipient's name, date of birth/age, diagnosis, the tissue-receiving surgeon’s name, and the date and location of the transplant surgery. The Eye Bank keeps the records of donors and recipients confidential and privileged, as well as the communications between them [17,20,22].

### The importance of eye donation

Corneal blindness is a major cause of visual impairment globally, often resulting from infections, injuries, degenerative diseases, or inherited conditions. Unlike many other types of blindness, corneal blindness can be effectively treated with corneal transplantation, which replaces diseased tissue with healthy donor tissue. However, the demand for corneas far exceeds the available supply, making eye donations essential [17,20,22].

### Challenges faced by the eye banks

- Shortage of donors: Despite ongoing awareness efforts, the number of eye donors does not meet the growing demand for corneal transplants. Cultural, religious, and personal beliefs about eye donation, as well as limited awareness, often hinder donor recruitment [9,17,20,22].
- Technological and logistical issues: Maintaining the quality and safety of donated tissues requires advanced technology and skilled personnel. Additionally, logistical challenges, such as coordinating the timely recovery and transport of tissues, can affect the availability of corneas [9,17,20,22].
- Regulatory and ethical concerns: Eye banks must navigate complex regulatory environments to ensure ethical practices in donation, processing, and distribution. Compliance with national and international standards is crucial for maintaining public trust and the integrity of the donation process [9,17,20,22].

### Impact of eye banks on society

Eye banks have a profound impact on society by restoring sight to those suffering from corneal blindness, thereby improving their quality of life, enabling independence, and enhancing productivity. Successful corneal transplants can transform recipients’ lives, allowing them to return to work, pursue education, and participate fully in their communities [33].

### How to pledge for eye donation

Becoming an eye donor is a simple yet impactful decision that can change lives. Individuals can pledge their eyes by registering with local eye banks or signing up through organ and tissue donation registries. It is also important to inform family members of the decision, as their consent will be required at the time of donation [22].

### Corneal transplantation

Corneal transplantation/corneal grafting/keratoplasty includes various surgical techniques used to restore vision. Globally, approximately 185,000 corneal transplants are performed annually. Countries such as the United States, Lebanon, and Canada lead in the number of procedures, with the United States alone performing 47,000 surgeries each year [3,7,38].

Corneal transplants are recommended for several purposes, including [3,7,38]:

- Optical: To enhance Visual Acuity in cases such as pseudophakic bullous keratopathy, keratoconus, corneal degeneration, keratoglobus, dystrophies, or scarring caused by keratitis and trauma.
- Tectonic/Reconstructive: To maintain corneal structure and integrity, particularly in conditions like stromal thinning or descemetoceles, such as those following corneal perforation.
- Therapeutic: To remove infected corneal tissue unresponsive to medical treatment, like antibiotics or antivirals.
- Cosmetic: To improve the appearance of eyes affected by corneal scarring.

Several conditions can lead to corneal damage requiring keratoplasty, such as [3,7,38]:

1. Keratoconus: A condition where the cornea becomes cone-shaped.
2. Fuchs’ Dystrophy: Dysfunction of corneal endothelial cells.
3. Infections or Injuries: Scarring or ulceration of the cornea due to trauma or infection.
4. Bullous Keratopathy: Swelling of the cornea due to endothelial cell failure.
5. Post-Surgical Damage: Corneal complications following eye surgeries.

### Types of corneal transplant surgeries

These techniques continue to evolve, offering patients improved outcomes and quicker recoveries. The anatomical structure of the cornea is defined for the different procedures applied in Keratoplasty [3,8].

**Fig. 1:**
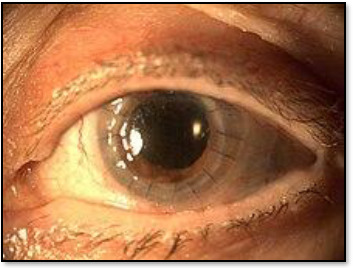
Full-thickness penetrating keratoplasty showing sutures’ position around the periphery of the grafted cornea [3,7]

1. Penetrating Keratoplasty (PK)
2. Deep Anterior Lamellar Keratoplasty (DALK)
3. Endothelial Keratoplasty (EK)

1. Penetrating keratoplasty (PK):
  It is also known as a full-thickness corneal transplant. The donor’s cornea replaces the entire cornea. PK has a longer healing time than other transplants and a slightly higher risk of graft rejection because of the body’s immune response. An eyelid speculum is placed to keep the lid open, a trephine removes a circular disc of the cornea of the donor, and then a second one is used to remove a similar-sized portion of the recipient’s cornea. The donor tissue is secured with sutures. Antibiotic eyedrops are applied before the patching [3,7] (**Fig. 1**).
2. Lamellar keratoplasty
  This partial-thickness transplant, also referred to as Anterior Lamellar Keratoplasty (ALK), a major advancement introduced in 2007, allows a single donor cornea to benefit two recipients and addresses conditions such as anterior corneal opacities, scars, and ectatic diseases. Superficial Anterior Lamellar Keratoplasty (SALK) replaces damaged epithelium. Deep Anterior Lamellar Keratoplasty (DALK) is used to replace the epithelium, Bowman’s layer, and stroma while preserving the endothelium. Deep Anterior Lamellar Keratoplasty (DALK) is commonly used for keratoconus. The advantages of the technique include shorter healing time and a lower risk of rejection compared with Penetrating Keratoplasty (PK), while maintaining the tectonic integrity of the eye [4,3,7,39].
3. Endothelial keratoplasty
  Introduced by Gerrit Melles et al. in 1998, EK (Endothelial Keratoplasty is a more precise form of posterior lamellar keratoplasty (PLK) as it covers a broad area of corneal surgery. Targeting damage to the corneal endothelium, which can cause corneal swelling, pain, and vision loss. While effective for endothelial failure, it does not address corneal scarring, thinning, and irregular surfaces. Two types of endothelial keratoplasty are the following [37,38,40,41]:
  - Descemet’s Stripping Endothelial Keratoplasty (DSEK) and Descemet’s Stripping Automated Endothelial Keratoplasty (DSAEK) apply a thicker graft, making it easier to transplant. The tissue is either prepared before keratoplasty or obtained ready-made for the procedure, which was first developed by the Ocular System organisation in 2005.
  - Descemet’s Membrane Endothelial Keratoplasty (DMEK) was introduced in 2006, which transplanted only the endothelium and Descemet’s membrane for better precision. It uses a thinner graft for quicker visual recovery and a lower risk of rejection. Minor automated variations for posterior lamella dissection are named Descemet’s Membrane Endothelial Keratoplasty (DMAEK) [42].

The procedure begins with a small incision to insert the endothelial graft and is often secured with an air bubble. Patients are advised to rest face-up for a few days to ensure proper graft positioning. In 2001, Mark Terry used viscoelastic rather than air bubbles and named it Deep Lamellar Endothelial Keratoplasty (DLEK). The graft self-adheres within a short time as the air bubble is absorbed.

In 2004, Melles refined the technique, replacing only the endothelium and Descemet membrane (DM) without stromal dissection, a procedure known as Descemet Stripping Endothelial Keratoplasty (DSEK). Subsequently, microtome-based automation led to the development of Descemet’s Stripping Automated Endothelial Keratoplasty (DSAEK) [43].

### Procedure of corneal transplant

During corneal transplantation, the use of 5% povidone-iodine combined with Amikacin and Gatifloxacin eye drops significantly reduces the risk of infection in the donor cornea. These antimicrobial agents provide critical protection against various pathogens and support the safety and success of the procedure [17].

Sedation or general anaesthesia is administered, with the procedure lasting about two hours. Patients may experience redness, irritation, and light sensitivity. Follow-up visits are required within 24–48 hours, and medications such as antibiotics and corticosteroids are used to control infection and inflammation. For endothelial transplants, patients must remain face-up for a few days. In full-thickness transplants, irregular astigmatism may necessitate refractive correction [44].

### Precautions and risk of graft rejection

Fitness against general health, avoid blood thinners, and driving. Medically warning signs are redness, pain, light sensitivity, and blurred vision. In many instances, an implanted cornea does not achieve its ideal curvature, leading to astigmatism; therefore, refractive correction is needed, as in cases of macular degeneration, glaucoma, and diabetic retinopathy [45].

The graft rejection rate is about 10% of cases and happens years after transplantation. Early failure may result from excessively tight sutures, severe dry eye, or other infections. The complications include Pannus formation, raised IOP, irregular corneal curvature, graft detachment, and retinal detachment. Usually, steroids and other interventions can help mitigate immune rejection [3,8].

- Immunity:
  Immuno-suppressants such as Cyclosporine A, Tacrolimus, Mycophenolate mofetil, Sirolimus, and Leflunomide are commonly used to prevent graft rejection in keratoplasty. However, determining the most effective option remains challenging. Studies indicate that systemic mycophenolate mofetil is associated with more frequent adverse effects than topical agents such as Cyclosporine A and Tacrolimus, which are generally better tolerated [46].
- Infection:
  The cornea’s avascular nature results in slow healing and increases the risk of infection from various microorganisms. Prophylactic antibiotics are commonly used to reduce the risk of infections; thorough screening for viral pathogens using antibody and nucleic acid tests has made infections rare in practice [47].

### Innovative corneal resources

1. Bioengineered cornea (stem cell)
  It offers a promising alternative for patients who cannot achieve vision restoration through traditional corneal transplants. Stem cells harvested from a healthy cornea are cultivated in the lab to form multiple layers of cells, which are then transplanted onto the damaged cornea. The stem cells maintain their ability to replicate and differentiate, helping prevent disease recurrence and ensuring long-term stability [48].
2. Biosynthetic cornea
  On August 25, 2010, researchers from Canada and Sweden published findings from the first clinical trial involving biosynthetic corneas. The Boston Keratoprostheses and osteo-odonto-keratoprosthesis (OOKP) have a polymer optic and a rim that serves as the interface with the recipient cornea. These devices are reported to be the most successful alternative to the cornea [49].

However, post-implantation of such devices showed improved vision, with corneal cell and nerve regeneration. While promising, outcomes remain inferior to those of human donor corneas, and the method remains experimental and is therefore estimated to be reserved for end-stage treatment [49].

Synthetic corneas, made from biocompatible materials, are used in cases with a high risk of graft failure. They consist of a peripheral skirt with a transparent central region, typically made of poly (2-hydroxyethyl methacrylate) (PHEMA) [50].

**Fig. 2:**
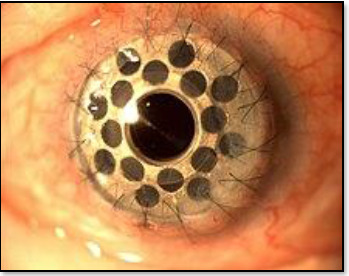
Synthetic Boston keratoprosthesis made up of phema material, replacing the natural cornea [50]

**Fig. 3:**
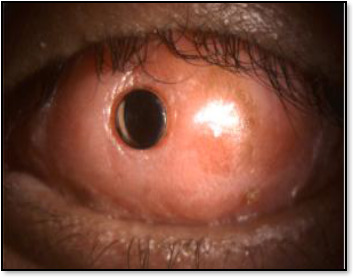
Osteo-odento-keratoprosthesis, an alternative to the artificial cornea [53]

- Boston Keratoprosthesis is the Kpro type-1 titanium keratoprosthesis, the most widely used synthetic cornea, developed at the Massachusetts Eye and Ear Infirmary. By 2008, more than 900 procedures had been performed worldwide [50] (**Fig. 2**).
- AlphaCor is an FDA-approved synthetic cornea with a diameter of 7.0 mm and a thickness of 0.5 mm [51].
- Osteo-Odonto-Keratoprosthesis (OOKP) is also known as tooth-in-eye surgery, a biological haptic designed by Strampelli and later on, modified Odonto-Keratoprosthesis (MOOKP) by Falcinelli is a highly complex, multi-step procedure for cases of bilateral corneal blindness or end-stage ocular surface disease. A tooth with surrounding alveolar bone is extracted from the donor, and a Poly-Methyl-Methacrylate (PMMA) optical cylinder is attached to create an implant. The procedure involves two stages, 4–5 months apart, each lasting 6–8 hours. It is primarily used to treat severe dry eye caused by Stevens-Johnson Syndrome, chemical burns, or failed stem cell transplants. Though rare and specialized, it is the gold standard for end-stage ocular surface diseases when other treatments fail (**Fig. 3**) [52-53].

### Refractive error management after corneal transplant

Corrective glasses or contact lenses are often needed after corneal transplants, especially following PK, which can cause irregular astigmatism. In cases like Penetrating Keratoplasty (PK) or Deep Anterior Lamellar Keratoplasty (DALK), rigid gas-permeable lenses may be required postoperatively. While these are not substitutes for keratoplasty, these options can help delay the need for such procedures [3,8].

- Contact Lenses: Specialized contact lenses can be used to postpone or potentially eliminate the need for a corneal transplant [53].
- Phototherapeutic Keratectomy (PTK): employed for superficial corneal surface disorders. It uses an excimer laser and a modulating agent to smooth irregularities [53].
- Intrastromal Corneal Ring Segments (ICRS): These semi-circular crescent-shaped rings are inserted into the stromal layer of the cornea, one on each side of the pupil. They flatten the cornea to address astigmatism or myopia and are commonly used to treat keratoconus [53].

## Discussion

Eye banking and various corneal replacement options are helping rehabilitate visual impairment; recent advancements, such as the EndoSaver, have made the insertion process more efficient. Descemet’s Membrane Endothelial Keratoplasty (DMEK), the latest technique, is available in countries such as the U.K. under the National Health Service (at Royal Shrewsbury Hospital, Calderdale and Huddersfield NHS Trust, and Worthing Hospital).

Modern keratoplasty techniques have transitioned from traditional blades to high-speed lasers, enhancing surgical precision, accelerating healing, and improving visual recovery.

Notable advancements include:

1. Amnitrans Eyebank (Rotterdam, The Netherlands): Since 2004, providing pre-cut donor corneas for advanced procedures like Descemet’s Stripping Endothelial Keratoplasty (DSEK), Descemet’s Stripping Automated Endothelial Keratoplasty (DSAEK), Femtosecond laser Semi-assisted Descemet Stripping Endothelial Keratoplasty (FS-DSEK), and Descemet’s Membrane Endothelial Keratoplasty (DMEK).
2. SightLife (Seattle, USA): Introduced femtosecond laser technology (2007) for a customized device of donor corneal tissue.

Advances in immunology, meticulous procedures, and tissue banking have collectively influenced corneal transplantation. Over many years, specific endothelial replacement procedures have transformed the field.

Selective lamellar transplantation has Replaced Penetrating Keratoplasty (PKP) for many indications, such as keratoconus and Fuchs endothelial dystrophy. By 2022, the rates of Replaced Penetrating Keratoplasty (PKP), Descemet’s Stripping Automated Endothelial Keratoplasty (DSAEK), and Descemet’s Membrane Endothelial Keratoplasty (DMEK) policy in the U.S. were nearly equal, highlighting the shift toward lamellar techniques due to better outcomes and fewer complications [43].

## Conclusion

Eye banks are essential in the fight against corneal blindness, providing a framework for gathering, preserving, and using donor tissue. Through their efforts, countless people can regain their vision and lead fulfilling lives. Nonetheless, the progress of eye banks relies on public awareness, donor interest, and ongoing support for their core work. By deciding to give eyes, people can leave an enduring tradition of sight, offering trust and vision to those in need. The headway of the various strategies is likewise simpler, leading to faster recovery and better outcomes in keratoplasty; the use of a single cornea for both recipients is also an incredible achievement, given the slow pace of donor cornea availability.

Modern keratoplasty techniques have transitioned from traditional blades to high-speed lasers, enhancing surgical precision, accelerating healing, and improving visual recovery. Among these advancements, lamellar keratoplasty methods enable a single donor cornea to benefit two recipients. Techniques such as Deep Anterior Lamellar Keratoplasty (DALK) and Descemet’s Stripping Endothelial Keratoplasty (DSEK) are broadly used. Descemet’s Membrane Endothelial Keratoplasty (DMEK) is a recent technique that offers improved outcomes by specifically targeting the damaged endothelial layer.

### Recommendation

1. Strengthen Eye Banking Infrastructure by upgrading facilities and establishing modern preservation and distribution systems in developing countries.
2. Enhance Public Awareness and Donor Education to overcome socio-cultural, religious, and ethical barriers to corneal donation.
3. Promote Access to Advanced Keratoplasty Techniques through equitable distribution of modern technologies and surgical training.
4. Support Training and capacity-building programs for ophthalmic surgeons and eye bank professionals.
5. Encourage Region-Specific Research on long-term outcomes, socio-cultural influences, and donation barriers.
6. Foster International Collaborations to facilitate knowledge exchange, technical support, and cross-border donor tissue sharing.

### Limitations

This study is based on a narrative review of the existing literature, clinical reports, and institutional guidelines, and does not include primary clinical data or prospective patient studies. The availability and implementation of advanced keratoplasty techniques and eye banking services vary significantly between high-income and low- to middle-income countries, potentially limiting the applicability of the findings across all regions. Disparities in healthcare infrastructure, economic resources, and government-supported ophthalmic programs influence the outcomes and operational capabilities of eye banks. Yet, these issues are not uniformly addressed in the available literature.

Moreover, the review highlights the challenges related to donor shortages, logistical hurdles, and ethical concerns; however, the underlying socio-cultural, religious, and legislative factors that impact corneal donation rates, particularly in Africa and Asia, remain insufficiently documented and warrant further investigation. Data on long-term patient outcomes following newer keratoplasty techniques and the widespread adoption of technologies such as femtosecond lasers are limited in resource-constrained settings. This limitation restricts a comprehensive global comparison of clinical success rates and patient-reported visual outcomes. Future studies incorporating large-scale, multi-center, and region-specific clinical data are recommended to address these gaps.
